# Application of Benzo(*a*)pyrene and Coal Tar Tumor Dose–Response Data to a Modified Benchmark Dose Method of Guideline Development

**DOI:** 10.1289/ehp.6427

**Published:** 2004-07-15

**Authors:** D. James Fitzgerald, Neville I. Robinson, Beverly A. Pester

**Affiliations:** ^1^Environmental Health Service, Department of Health, Adelaide, South Australia, Australia; ^2^Division of Mathematical and Information Sciences, Commonwealth Scientific and Industrial Research Organisation, Urrbrae, Adelaide, South Australia, Australia; ^3^School of Chemistry, Physics and Earth Sciences, Flinders University, Bedford Park, South Australia, Australia

**Keywords:** benzo(*a*)pyrene, cancer risk assessment, dose-response modeling, modified benchmark dose method, PAH, 7*H*-benzo(*c*)fluorene, soil carcinogens

## Abstract

Assessment of cancer risk from exposure to polycyclic aromatic hydrocarbons (PAHs) has been traditionally conducted by applying the conservative linearized multistage (LMS) model to animal tumor data for benzo(*a*)pyrene (BaP), considered the most potent carcinogen in PAH mixtures. Because it has been argued that LMS use of 95% lower confidence limits on dose is unnecessarily conservative, that assumptions of low-dose linearity to zero in the dose response imply clear mechanistic understanding, and that “acceptable” cancer risk rests on a policy decision, an alternative cancer risk assessment approach has been developed. Based in part on the emerging benchmark dose (BMD) method, the modified BMD method we used involves applying a suite of conventional mathematical models to tumor dose–response data. This permits derivation of the average dose corresponding to 5% extra tumor incidence (BMD_0.05_) to which a number of modifying factors are applied to achieve a guideline dose, that is, a daily dose considered safe for human lifetime exposure. Application of the modified BMD method to recent forestomach tumor data from BaP ingestion studies in mice suggests a guideline dose of 0.08 μg/kg/day. Based on this and an understanding of dietary BaP, and considering that BaP is a common contaminant in soil and therefore poses human health risk via soil ingestion, we propose a BaP soil guideline value of 5 ppm (milligrams per kilogram). Mouse tumor data from ingestion of coal tar mixtures containing PAHs and BaP show that lung and not forestomach tumors are most prevalent and that BaP content cannot explain the lung tumors. This calls into question the common use of toxicity equivalence factors based on BaP for assessing risk from complex PAH mixtures. Emerging data point to another PAH compound—7*H*-benzo(*c*)fluorene—as the possible lung tumorigen.

Polycyclic aromatic hydrocarbons (PAHs) are found at a variety of contaminated sites throughout the world from industries such as coal gasification, coke production, aluminum production, iron and steel foundries, and creosote and asphalt production. Some PAHs, for example, the well-studied benzo(*a*)pyrene (BaP), are mutagenic and carcinogenic in experimental animals and probably in humans also [Boffetta et al. 1997; International Agency for Research on Cancer (IARC) 1987; Rubin 2001]. Therefore, health risk assessment of PAHs with a view to setting acceptable levels in contaminated soil is an important challenge for regulatory toxicologists.

Various methods are employed by agencies to estimate the risk posed by a certain level of soil contaminant, all of which have advantages and disadvantages. Threshold methods seek to determine a threshold below which no adverse effects are expected and that yield values such as the tolerable daily intake (TDI) or reference dose (RfD). These methods have the disadvantage that they hinge upon the no observed adverse effect level (NOAEL), which must be one of the chosen exposure levels in a toxicologic study. This exposure level is unlikely to be the actual threshold no effect level. For setting guideline levels based on cancer risk, the linearized multistage (LMS) model is used by the U.S. Environmental Protection Agency (EPA); this model assumes that the dose–response curve is linear in the low-dose region of the curve and that no threshold exists. This assumption has the disadvantages of not taking into account the complexities of the carcinogenic process and of not accommodating the possibility that the dose–response data may be best explained by a curve that is nonlinear in the low-dose region. In addition, the LMS approach requires a societal judgment on what constitutes an “acceptable” level of risk. Use of the LMS method results in the most conservative regulatory guidelines.

An alternative approach to the preparation of regulatory guidelines is the benchmark dose (BMD) method to model toxicologic end points. This method uses conventional mathematical models to obtain dose–response curves; that is, it does not assume linearity in the low-dose region. The BMD approach has been developed particularly by Crump (1984, 1995) and the U.S. EPA (1995). The current U.S. EPA default approach is to calculate the 95% lower confidence limit on a dose associated with a 10% extra tumor risk level (U.S. EPA 2003). However, the disadvantage of this method is that it applies a statistically derived 95% lower confidence limit on the dose–response curve that may not be valid for the small data sets often encountered in toxicology studies.

The National Health and Medical Research Council (NHMRC) of Australia embarked on a project to identify a cancer risk assessment process that avoided the extreme conservatism inherent in the assumption of low-dose linearity but that used any available dose–response data. The NHMRC Technical Working Party on Carcinogenic Risk Assessment for Soil Contaminants developed the modified BMD method (NHMRC 1999). This approach combines toxicologic dose–response data (usually from animal studies) and conventional mathematical models to generate dose–response curves for the chemical in question, even in the subexperimental region, and does not assume a linear relationship in this region. The approach avoids the conservatism of other BMD models by relying on best-fit modeling rather than 95% lower confidence limits on dose. For the various models applied, the technique determines an average dose at which 5% extra risk is incurred (BMD_0.05_); this level of risk was chosen because it is near the lower limit of responses that can be experimentally measured. Modifying factors reflecting the degree of uncertainty in extrapolating from animal exposure are then applied to yield a guideline dose for human exposure.

The purpose of this study was to use the modified BMD method to construct tumor dose–response curves for BaP using data from a recently published 2-year feeding study on female B6C3F1 mice (Culp et al. 1998). Previous rodent BaP feeding studies were also evaluated but either lacked sufficient data points or exposure times (Brune et al. 1981; Neal and Rigdon 1967) or suggested lesser sensitivity (Kroese et al. 2001). We used the BMD obtained from the Culp et al. (1998) data set to calculate a guideline value for BaP in soil.

The recent BaP bioassay (Culp et al. 1998) also examined the tumor response of female B6C3F1 mice to two coal tar mixtures. Although data generated would be reasonably assumed to be useful in assessing risk from exposure to complex mixtures, such data reveal some unresolved issues. These relate primarily to the difficulties of the simplistic BaP-equivalence approach of PAH additivity in the mixture, and the emerging notion that perhaps a PAH other than BaP ought to be the risk driver in these mixtures. This is further discussed in the present article.

In conducting this exercise, it has been necessary to adhere to the process set out in the nationally developed modified BMD method document (NHMRC 1999). However, as with any emerging field, refinements will be proposed over time that will decrease uncertainties in this risk assessment approach.

## Materials and Methods

### Dose calculation.

Culp et al. (1998) reported their dose data as dietary concentrations. To permit us to convert dietary concentrations to average daily doses on a body weight basis, S. Culp (National Center for Toxicological Research, Jefferson, AR, USA) provided information on the average amount of food consumed per animal per day and the average animal body weights. We calculated average doses in units of milligrams per kilogram per day for each of the 12 dose groups every 4 weeks until the end of the study, or until all animals were removed from the study in a particular dose group. These doses were then averaged to obtain an “average lifetime dose” for each group, as presented in Table 1. The doses for the coal tar mixtures are also given in BaP equivalents for comparison purposes. These BaP equivalents were calculated using previously published toxicity equivalence factors (TEFs) for PAH mixtures (Fitzgerald 1998).

Culp et al. (1998) reported results for several types of tumors induced by BaP and two coal tar mixtures. For BaP, forestomach tumors proved to be the most sensitive end point (Table 1), and these dose–response data are used here to determine a guideline value. In the case of the coal tar mixtures, lung tumors were shown to be the most sensitive end point (Table 1).

### Mathematical modeling.

The NHMRC Technical Working Party document on the modified BMD method (NHMRC 1999) requires the construction of dose–response curves by fitting dose–response data with a suite of mathematical models. A suite is used to overcome bias when using a single model that is attempting to simulate an underlying but unknown model. The models are parametric and are the cumulative probability distribution functions (cdfs) for the well-known Weibull, log normal (probit), log logistic, gamma (multi-hit), and linear exponential (single hit) distributions as well as the truncated logistic and truncated normal distributions. The NHMRC recommends use of the Weibull, probit, and linear exponential models as a default selection with the option of an expanded or alternative selection of models. The expanded set of seven models has three parameters to be found from the data (except for the linear exponential, with two) and includes the zero dose background response. We chose three-parameter models for parsimony and because data sets for carcinogens often consist of just three or four data points.

Dose–extra-risk curves are determined by transformation of dose–response curves in the following way. If the cdfs are represented by P*(*d*) for a dose *d*, such that P*(*d*) ranges from 0 at *d* = 0 to 1 for a very high dose, then the fitted models all have the form *P*(*d*) = *c* + (1 – *c*)P*(*d*), where *c* is the background response. Extra risk is then defined by *R* = [*P*(*d*) – *P*(0)] ÷ [(1 – *P*(0)] = P*(*d*). At *R* = 5%, the BMD_0.05_ for a particular model is that value of *d* such that P*(*d*) = 0.05. This value is then determined for each model. Results from any particular curve are discarded only if it is clear that the model does not fit. The NHMRC procedure (NHMRC 1999) uses the BMD_0.05_ as determined by each acceptable model and then arithmetically averages them. The details of calculation of the BMD_0.05_ using the maximum likelihood estimate (MLE) for fitting cdf values to the data are provided in the Supplemental Material available online (http://ehp.niehs.nih.gov/members/2004/6427/supplemental.pdf).

### Modifying factors.

To develop the guideline value from the BMD_0.05_ requires dividing the BMD_0.05_ by a modifying factor that takes into account interspecies extrapolation, intraspecies variability, the quality of the data set as a whole, the ability of the compound to induce malignant tumors, and the genotoxicity of the compound in question (NHMRC 1999).

### Modifying factors for BaP.

Table 2 lists the modifying factors established for BaP, the numerical range of the factors, and the factors proposed for use in this guideline value development. The development of modifying factors for BaP was previously discussed in the use of a preliminary BMD method to derive a guideline value for BaP (Fitzgerald 1998). The modifying factor of 6,000 is slightly altered here in light of the additional data obtained from the recent studies by Culp et al. (1998).

BaP exhibits high lipophilicity and is metabolized in all tissues studied, and its metabolites are potent gene and chromosome mutagens, suggesting that the response of humans to BaP is likely to be more similar to that of mice than a maximum (default) inter-species extrapolation factor of 10 would imply (i.e., there is no evidence indicating that humans could be 10 times more sensitive than mice to BaP carcinogenicity). Several *in vitro* studies of BaP metabolism, mutagenicity, and DNA adduct formation using human and animal cells or tissue components suggest that BaP is not more toxicologically active in human cells than in mouse cells (Hengstler et al. 1999; Hsu et al. 1987; Oesch et al. 1977; Roggeband et al. 1993). However, an exception to this is seen with comparative studies of mammary cells exposed to BaP (Hengstler et al. 1999). Given this, and the limitations of extrapolating from *in vitro* data, we propose a modifying factor of 5 for interspecies extrapolation.

The intraspecies variability factor is set at 10 because of the lack of human data available. The adequacy of database factor, whereby the better the quality of the relevant tumor studies the smaller the factor, is given a value of 2 to reflect a high degree of confidence. The study of Culp et al. (1998) extended over the lifetime of the animals and included a suitable number of dose levels. The malignancy of BaP in a range of tissues is well established and—together with the Culp et al. (1998) bioassay study in which BaP induced tumors in the esophagus, tongue, larynx, and forestomach—engenders a proposed modifying factor of 9. The maximum factor of 5 for genotoxicity was assigned because this property of BaP is well established and BaP is a potent mutagen.

Thus, the overall modifying factor is 5 × 10 × 2 × 9 × 5 = 4,500.

## Results

### BaP.

**Figure 1:**
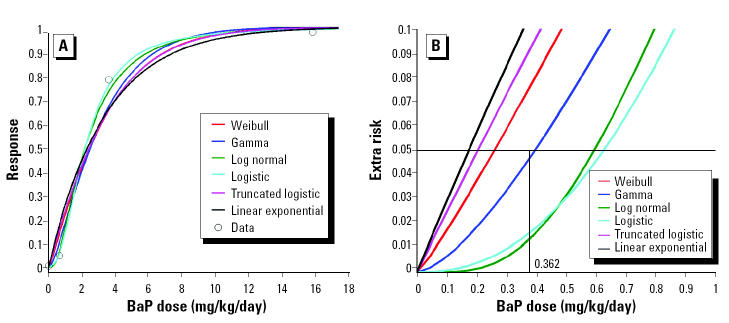
Suite of models fitted to BaP dose–response data (mouse forestomach tumors) reported by Culp et al. (1998). (*A*) MLE fitting of models except the truncated normal, which could not be fitted. (*B*) The extra-risk dose curves of (*A*) in the low-dose region around the 0.05 risk level and averaged dose at 0.362 mg/kg/day.

The fitting of forestomach tumor dose–response curves to the the BaP data of Culp et al. (1998) (Table 1) is shown in Figure 1. Figure 1A depicts the plotted models relative to the Culp et al. BaP data, and Figure 1B shows the extra risk–dose curves derived from them. The calculated value of 0.362 mg/kg/day for BMD_0.05_, as shown in the Supplemental Material (http://ehp.niehs.nih.gov/members/2004/6427/supplemental.pdf), is an average from six of the models. The excluded model is the truncated normal model because it could not be fitted to the data. This lack of fit occurs when the curves are “supra-linear” or nearly so, as is the case here. This may also occur with some data sets for the truncated logistic model.

### Development of a soil BaP guideline value from the BMD_0.05_.

Taking the BaP BMD_0.05_ of 0.362 mg/kg/day and applying the modifying factor of 4,500 yields the following guideline dose:


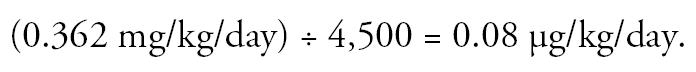


This yields the following maximum daily intakes (MDI) for adults (assuming 70 kg body weight) and children (assuming 13.2 kg for a 2-year-old child):


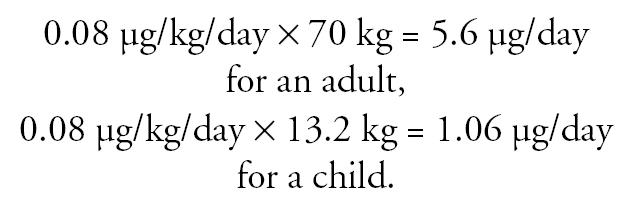


These MDIs represent the total daily BaP intake that should not be exceeded in order to safeguard human health. Some of this intake is assumed to come from food; consequently, the TDI from soil is calculated to be the MDI minus the intake from food, divided by 2 for a measure of safety and to allow for some exposures via air and water (Fitzgerald 1998).

In a previous BaP guideline value calculation (Fitzgerald 1998), a U.S. EPA upper estimate for BaP intake in food of 1.6 μg/day (U.S. EPA 1980) and a U.K. estimate of a child’s BaP intake being 40% of an adult’s intake (RPS 1995) were used. If we used these same values, the allowable daily intake for BaP from soil would be (5.6 – 1.6) ÷2 = 2.0 μg for adults and (1.06 – 0.64) ÷2 = 0.21 μg for children. Based on an assumed adult soil ingestion rate of 25 mg/day [Australia and New Zealand Environment Conservation Council (ANZECC) and NHMRC 1992], a BaP soil guideline value would be


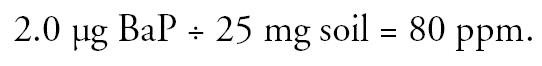


For children, an assumed soil ingestion rate is 100 mg/day (ANZECC and NHMRC 1992); thus, a BaP soil guideline value would be


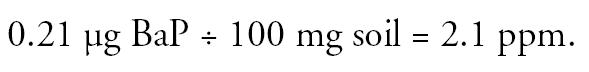


One of the key data sets in this approach is the estimate for daily dietary BaP intake. Better estimates for intake than those used above may be obtained from recent data from a U.S. study of 200 food items and 228 subjects (Kazerouni et al. 2001), which indicated that all adults in the study consumed < 0.16 μg BaP/day. Applying this to the above method, the allowable daily BaP intake from soil would be (5.6 – 0.16) ÷2 = 2.72 μg for adults and (1.06 – 0.06) ÷2 = 0.50 μg for children. Further, a BaP soil guideline based on adult soil ingestion would be


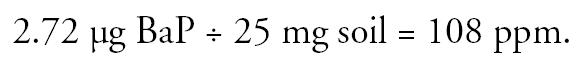


For children, the calculation would be


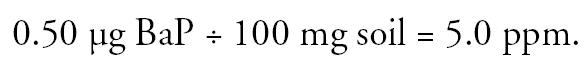


As previously suggested (Fitzgerald 1998), the least value of such calculations is proposed as the BaP soil guideline value, in this case, 5 ppm.

### Coal tar mixtures.

Using the coal tar doses from the cancer bioassay study of Culp et al. (1998) (Table 1) to develop a guideline value is complicated by numerous factors. There is insufficient toxicologic information available on coal tar mixtures to confidently establish defensible modifying factors. In addition, there are no published MDI values for coal tar (or PAH) mixtures and no figures available on average coal tar (or PAH) intake from diet. Even if these figures were available, mixtures of coal tars and their bioavailability differ according to their source, the soil type, and degree of “aging” in the environment (Abdel-Rahman et al. 2002; Bordelon et al. 2000; Reeves et al. 2001). Consequently, any guideline value developed from cancer bio-assay data on a particular coal tar mixture may not apply to subsequently encountered coal tar mixtures.

Instead, a pragmatic approach commonly taken with PAH mixtures is to calculate the BaP equivalence dose, based on TEFs with BaP as the reference carcinogen (Boström et al. 2002; Fitzgerald 1998). For the present coal tar mixtures, this addition of BaP equivalents using previously proposed equivalence factors (Fitzgerald 1998) resulted in BaP equivalence doses approximately twice the actual BaP concentrations (Table 1). These calculations showed < 30% variance from BaP equivalence doses generated by two other TEF schemes (Larsen and Larsen 1998; Nisbet and LaGoy 1992). Not previously considered for TEFs were the naphthalene derivatives 1-methyl-naphthalene and 2-methylnaphthalene, which were prominent PAHs in the coal tar mixtures (Culp et al. 1998). Available toxicity data were limited, and TEFs of 0.001 were assigned to both isomers.

The most sensitive tumorigenic response to the coal tar mixtures was with lung tumors (Culp et al. 1998). Preliminary modeling of BaP equivalence doses and lung tumor data of coal tar exposures (not shown) revealed non-simple fits and considerable variability between the mixtures. Further detailed analysis is beyond the scope of the present study.

## Discussion

The present study represents the first significant attempt to use the modified BMD method as developed in Australia for generating guideline values for environmental carcinogens. The present program focuses on BaP as the key surrogate for PAHs and builds on preliminary work in this area (Fitzgerald 1998). With this approach, we propose a BaP soil guideline value of 5 ppm. This would represent a significant departure from the current Australian soil guideline for BaP of 1 ppm that was based on consideration of proportionality of dietary BaP and related cancer risk derived from U.S. EPA LMS modeling (Fitzgerald 1991; National Environment Protection Council 1999).

In the absence of human data, the described method has employed experimental animal data. The BMD_0.05_ of 0.362 mg BaP/kg/day we used is considered a refinement of the 0.815 mg BaP/kg/day BMD_0.05_ determined in previous work, employing the MLE method with quantal Weibull and polynomial regression modeling of earlier bioassays (Fitzgerald 1998; Neal and Rigdon 1967). Although Neal and Rigdon’s data set was the best BaP tumor dose response available at the time and includes more groups in the low-dose region than does the data set of Culp et al. (1998), we consider it to be less suitable for BMD development principally because of the less-than-lifetime BaP exposures (3–6 months vs. 24 months).

Although the use of computer-based modeling in guideline development is quite sophisticated and reasonably defensible scientifically, the component of guideline value derivation that involves modifying factors is probably the most subjective part of the entire process. Nonetheless, such factors are used routinely in regulatory toxicology and are often termed uncertainty factors or safety factors. For some of these, for example, default interspecies extrapolation and intraspecies variability, there are empirical data to indicate a fair degree of confidence that they are not unreasonable although likely to be conservative (Dourson et al. 1996; Fitzgerald 1993; Lewis et al. 1990). Where information exists to allow a factor other than the default, for example, comparative toxicokinetic data or, as in the present case, a range of intuitive arguments around interspecies BaP extrapolation, a nondefault factor can be used.

For the “safety factor” portion of the overall modifying factors, namely, database adequacy, malignancy, and genotoxicity, judgment is somewhat subjective. Nonetheless, the suggested approach is based on internationally used assessment methods [NHMRC 1999; World Health Organization (WHO) 1994].

A further variable of the guideline value equation that has a major bearing on the outcome is the estimate of daily dietary BaP intake. Recent data indicating that BaP intake may be decreasing over time (Kazerouni et al. 2001; U.S. EPA 1980) could perhaps be explained by the stricter emission controls on industries that release PAHs; reduced PAHs in air pollution would mean reduced deposition on plants and reduced uptake by farm animals (Kazerouni et al. 2001).

The final variable affecting the guideline value generation is that of soil ingestion rate. For the present study, we used daily rates of 25 mg for adults and 100 mg for children because they are generally adopted by regulatory toxicologists in Australia (ANZECC and NHMRC 1992). However, we recognize that these values may be different in other countries (Gargas et al. 2000), although the intake used for children is similar to 95th percentile estimates determined recently for children residing near a U.S. Superfund site (Stanek and Calabrese 2000).

**Figure 2:**
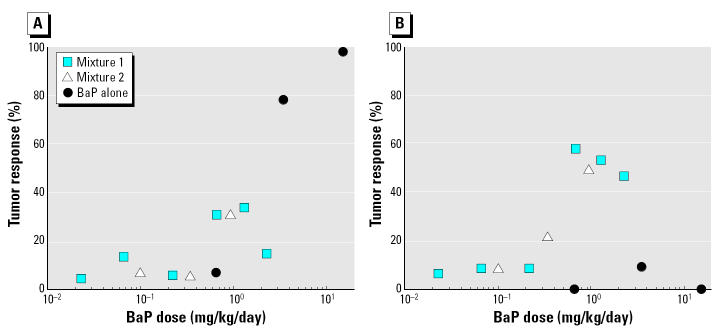
Comparison of dose responses for tumors reported by Culp et al. (1998), plotted for BaP alone and BaP content of coal tar mixtures. (*A*) Forestomach tumors. (*B*) Lung tumors.

Potentially the most significant aspect of the data of Culp et al. (1998) is that concerning tumor responses to dietary coal tar mixtures in which the tumor profile was quite different from that with BaP, both qualitatively and quantitatively. Of particular note was the finding that, purely in terms of concentration, the BaP in the mixtures could explain the forestomach tumors induced by the mixtures but could not explain the lung tumors (Figure 2); BaP alone was a weak inducer or noninducer of lung tumors at the doses tested. Such preferential induction of lung tumors in mice by a PAH mixture compared with BaP has been previously reported (Weyand et al. 1995).

Speculatively, one may propose either that the action of BaP (or BaP equivalents) is synergized in the mixture milieu in a way that selectively induces lung tumors, or that some other component of the mixture is tumorigenic in the mouse lung. The latter notion, together with indication of a non-BaP compound interacting with lung DNA (Culp et al. 2000; Goldstein et al. 1998; Weyand and Wu 1995), has led to the finding that the causative agent may be 7*H*-benzo(*c*)fluorene (BcF) (Goldstein 2001; Koganti et al. 2000, 2001) and that *in vivo* bioavailability and metabolism of this PAH are probably much greater than for BaP (Koganti et al. 2001). Recent evidence further points to dihydrodiol and diol epoxide metabolites of BcF as being the proximate and ultimate carcinogenic moieties, respectively, that bind to mouse lung DNA (Wang et al. 2002). Recent studies have also examined separately the lung carcinogenicity of BcF (about 9 mg/kg/day) and equimolar BaP (about 10 mg/kg/day) given in the diet of lung-tumor–susceptible female A/J mice over 260 days (Weyand EH, personal communication; Weyand et al. 2002). The data showed that BcF increased the prevalence of mice with lung tumors (from 77% for BaP to 100%) but, most significantly, increased the multiplicity of lung tumors 33-fold (Weyand et al. 2002). This suggests that for future PAH risk assessments and setting of regulatory guidelines, more consideration of BcF levels will be needed, as well as some rethinking of the prominence afforded BaP and associated TEFs in current regulatory science (Goldstein 2001). Culp et al. (1998) did not report on BcF levels in the coal tar mixtures used in their studies.

A further possible consideration stems from the notion that lung tumorigenesis ought to be the cancer risk assessment driver for ingested PAH mixtures. That is, because lung tumorigenesis is the risk assessment driver for air/PAH inhalation, then evaluation of soil particle inhalation should be contemplated. However, mitigating against this are recent data suggesting that the deposition efficiency of airborne soil particles in the tracheobronchial and pulmonary regions of the lung is very low (Khalili and Thomas 2001).

The BaP forestomach tumor data from Culp et al. (1998) have been used to revise the U.S. EPA LMS cancer slope factor for BaP (Gaylor et al. 1998, 2000). Also, risk assessors have proposed BaP soil guideline values using TDI based on the LMS paradigm and “acceptable” lifetime cancer risk estimates (Boyd et al. 1999). It is beyond the scope of this article to examine such approaches. However, the present BMD method is a departure from LMS that does not operate on an assumption of low-dose linearity or attempt any policy decision on acceptable human population cancer risk. Instead, it makes fuller use of all the tumor dose–response data and is based on a more realistic central estimate.

## Conclusion

We have proposed a guideline value for BaP in soil using a modified BMD method developed within the Australian regulatory toxicology community. As now required by the Australian government health authorities, this work will be extended to include other carcinogens that exist in a range of environmental media. Further work may also examine the general validity of the safety factors employed and whether scientific uncertainty around the 5% extra risk starting point may be reduced.

## Figures and Tables

**Table 1:** Doses for coal tar mixtures and BaP administered for 2 years in the diet of B6C3F1 mice,*^a^* and tumorigenic responses in forestomach and lung.

					Mice with tumors/total mice	
Dose group	Concentration in diet (ppm)	Average lifetime dose (mg/kg/day)*^b^*	BaP equivalent dose (mg/kg/day)*^c^*	Actual BaP dose (mg/kg/day)	Forestomach*^d^*	Lung*^e^*
Coal	0	0	0	0	0/47	2/47
Tar	100	12.4	0.051	0.023	2/47	3/48
Mix 1	300	35.8	0.15	0.066	6/45	4/48
	1,000	121	0.49	0.222	3/47	4/48
	3,000	367	1.46	0.675	14/46	27/47
	6,000*^f^*	707	2.92	1.299	15/45	25/47
	10,000*^f^*	1,234	5.01	2.268	6/41	21/47
Coal	0	0	0	0	0/47	2/47
Tar	300	36.4	0.21	0.100	3/47	4/48
Mix 2	1,000	124	0.72	0.342	2/47	10/48
	3,000	339	1.97	0.936	13/44	23/47
BaP	0			0	1/48	5/49
	5			0.65	3/47	0/48
	25			3.5	36/46	4/45
	100*^f^*			15.3	46/47	0/48

a Details from Culp et al. (1998) and S.J. Culp (personal communication); dose groups included zero dose controls, and animals in all groups were dosed for 2 years from 5 weeks of age.

b From animal weight and food intake data, averaged over the study period (Culp SJ, personal communication).

c From PAH levels in Culp et al. (1998) and from TEFs in Fitzgerald [1998; BaP, 1; dibenz(*a*,*h*)anthracene, 4; benz(*a*)anthracene, 0.1; benzo(*b*)fluoranthene, 0.1; benzo(*k*)fluoranthene, 0.1; indeno[1,2,3-*c,d*]pyrene, 0.1; anthracene, 0.001; benzo(*g,h,i*)perylene, 0.1; chrysene, 0.1; acenaphthene, 0.001; acenaphthylene, 0.001; fluoranthene, 0.01; fluorene, 0.001; naphthalene, 0.001; phenanthrene, 0.001; pyrene, 0.001].

d Forestomach papillomas and carcinomas.

e Alveolar and bronchial adenomas and carcinomas.

f At these doses, all tumor-bearing animals died before the end of the 2-year exposure period.

**Table 2:** Modifying factors for BaP.*^a^*

Factor	Range of value	BaP value
Interspecies extrapolation	≤1–10	5
Intraspecies variability	1–10	10
Database adequacy	1–2 (high)	2
	3–7 (medium)	
	8–10 (low)	
Malignancy	3–10	9
Genotoxicity	1–5	5
Overall factor		4,500

a See NHMRC (1999) and Fitzgerald (1998).
